# Epicardial Adipose Tissue in Patients with Coronary Artery Disease: A Meta-Analysis

**DOI:** 10.3390/jcdd9080253

**Published:** 2022-08-08

**Authors:** Qingpeng Wang, Jiangyang Chi, Chen Wang, Yun Yang, Rui Tian, Xinzhong Chen

**Affiliations:** 1 Department of Cardiovascular Surgery, Union Hospital, Tongji Medical College, Huazhong University of Science and Technology, Wuhan 430022, China; 2 Department of Ultrasound Medicine, Union Hospital, Tongji Medical College, Huazhong University of Science and Technology, Wuhan 430022, China

**Keywords:** epicardial adipose tissue (EAT), coronary artery disease (CAD), thickness, volume, meta-analysis

## Abstract

Objective: The aim of this study is to assess the association between epicardial adipose tissue (EAT) and coronary artery disease (CAD) via meta−analysis. Methods: Specific searches of online databases from January 2000 to May 2022 were conducted. All observational studies evaluating the association between EAT and CAD in PubMed, Web of Science, and the Cochrane Library databases were screened. A meta-analysis was conducted following the Preferred Reporting Items for Systematic Reviews and Meta−Analyses guidelines (PRISMA). In total, 21 studies encompassing 4975 subjects met the inclusion criteria, including 2377 diagnosed and assigned as the CAD group, while the other 2598 were assigned as the non−CAD group. Subjects in the CAD group were further divided into the severe stenosis group (stenosis ≥ 50%, *n* = 846) and the mild/moderate stenosis group (stenosis < 50%, *n* = 577). Results: Both the volume and thickness of EAT in the CAD group were larger compared to the non−CAD group (*p* < 0.00001). In a subgroup analysis within the CAD group, the severe stenosis group had a larger volume and thickness with respect to EAT when compared to the mild/moderate group (*p* < 0.001). Conclusions: The enlargement of EAT presented in CAD patients with an association with CAD severity. Although limited by different CAD types and measuring methods for EAT, as well as a smaller sample size, our results suggest that EAT is a novel predictor and a potential therapeutic target for CAD.

## 1. Introduction

Coronary artery disease (CAD) is a cardiovascular disease caused by reduced blood flow in the coronary arteries [1,2]. The clinical manifestations of CAD include silent myocardial ischemia, angina pectoris, acute coronary syndromes (unstable angina pectoris, myocardial infarction), and sudden cardiac death [3]. Adipose tissue is the largest endocrine organ in the human body, which plays an essential role in the supply of energy [4,5,6]. Recently, the relationship between adipose tissue and CAD has gained increasing attention. Relevant studies may provide new approaches for the treatment of CAD. 

Epicardial adipose tissue (EAT) is a type of visceral adipose tissue surrounding the myocardium and visceral layer of the pericardium [7,8]. In distinct conditions, EAT can secrete pro- and anti-inflammatory factors (e.g., TNF-α, IL-6, adiponectin, and leptin) through paracrine or endocrine [9,10,11]. Evidence shows that EAT is involved in the local regulation of myocardial and coronary function by modulating lipid metabolism and energy homeostasis [12]. By regulating the release / uptake of free fatty acids (FFAs), EAT plays an important role in CAD by supporting the efficiency of myocardial glucose utilization [13,14]. Clinically, the volume and thickness of EAT have been measured by cardiac magnetic resonance imaging (MRI), computed tomography (CT) [15], and echocardiography (echo) [16]. Several studies have shown that enlarged EAT is associated with the occurrence and development of CAD [17], which was later termed a potential predictor of the disease [16,18,19,20,21]. According to existing research, we speculate that the enlargement of EAT has been gradually becoming one of the key risk factors for the development of CAD [12,20,22,23]. However, a more specific correlation between EAT and CAD has yet to be clearly studied.

In the past few years, several studies have reported abnormally enlarged EAT in CAD patients [24,25]. However, due to the lack of a larger sample size and potential confounding factors, including differences in the EAT measures and CAD grades in these studies, the strength of previous evidence is limited. Therefore, in order to provide a comprehensive overview of this issue, we conducted a meta-analysis to assess the relationship between EAT and CAD.

## 2. Methods

### 2.1. Literature Search and Selection

We conducted a comprehensive systematic literature search of online databases, including PubMed, Embase, Web of Science, and the Cochrane Library from January 2000 to May 2022. To identify and retrieve all potentially relevant articles regarding this topic, all combinations of the following search terms were included: (coronary artery disease OR CAD, myocardial ischemia OR ischemic heart disease) AND (epicardial fat tissue OR epicardial adipose tissue OR subepicardial adipose tissue OR subepicardial fat tissue). An additional manual search was performed by analyzing the reference list of original publications and review articles.

All the search results were evaluated according to the Preferred Reporting Items for Systematic Reviews and Meta-Analyses (PRISMA) statement [26]. The diagnostic criteria for coronary heart disease established by the World Health Organization are: at least 1 coronary artery stenosis ≥ 50% in the right coronary artery, left main trunk, anterior descending or circumflex artery and its main branches [27,28]. In the single−vessel disease group, coronary artery stenosis was ≥50%. The stenosis of any two coronary artery lesions in the double−vessel disease group was greater than or equal to 50%. In the multivessel disease group, the stenosis of 3 coronary artery lesions was greater than or equal to 50%. CAD is defined as the presence of stenosis of any severity in at least one coronary artery segment [29]. However, the definition of CAD severity varied across studies. For instance, one study defined the degree of coronary artery stenosis as mild (<50%), moderate (50–69%), and severe (≥70%) [30]. In other studies, severe stenosis was only considered when coronary angiography showed stenosis in any vessel exceeding 50% [31,32,33,34], 70% [35,36], or even 75% [37,38,39,40]. In this study, we found that when any coronary artery stenosis ≥ 50%, obvious symptoms, including chest pain and chest tightness, are recorded. These symptoms can arise during myocardial ischemia or hypoxia due to the narrowing of blood vessels, which causes serious damage to patients’ health and lives [41]. In order to include more reliable studies, we defined 50% stenosis as a cutoff point for the severity of coronary artery disease whereby ≥ 50% stenosis in any single vessel in a coronary angiography was considered to belong to the significant stenosis group and others (<50%) were considered to belong to the mild/moderate stenosis group. In addition, we also classified stable angina (SA) and unstable angina (UA) as being part of the mild/moderate stenosis group and non-ST-segment elevation myocardial infarction (NSTEMI) and ST-segment elevation myocardial infarction (STEMI) as being part of the severe stenosis group, in accordance with the previously published standard [41].

The thickness of EAT typically describes the vertical distance of the echo-transparent zone between the pericardium visceral layer and the ventricle epicardium, and the mean thickness was calculated using average measurements of 3 cardiac cycles [42,43]. The volume of EAT describes the volume between the pericardium and the ventricular wall, with attenuation ranging from −250 HU to −30 HU measured by CT. The sum of EAT volume was calculated by adding up EAT areas using transverse sections from the atrial appendage up to the apex, with 1.0 cm spacing between each image [44].

Therefore, the inclusion criteria for studies were as follows: (1) CAD patients were an experimental group and non−CAD patients were a control group; (2) the quantitative measurement of EAT volume or thickness by echocardiography (echo), cardiac magnetic resonance imaging (MRI), or CT; (3) studies comparing differences in EAT between CAD patients and non−CAD patients; (4) studies with a reported mean, standard deviation (SD), and recorded sample sizes for CAD and non−CAD patients; and (5) observational studies. Exclusion criteria: (1) experimental animal studies, reviews, or non-English literature and (2) studies that did not provide sufficient information about the dataset (mean, SD, and sample sizes).

### 2.2. Data Extraction and Quality Assessment

Data extraction and literature quality assessment were performed independently by 2 investigators (Q.W. and Y.Y.). A Microsoft Excel database was used to record all available information, including basic details such as the number of people, sex, age, period, the volume or thickness of EAT, the number and location of coronary stenosis, the method of measuring EAT, etc. For quality assessment of the included studies, continuous and observational studies were assessed using the Cochrane Handbook for Systematic Reviews of Interventions (version 5.1.0) and the modified Newcastle–Ottawa Scale (NOS), respectively. Any disagreements were resolved by another investigator (C.W.).

### 2.3. Statistical Analysis

A meta-analysis was performed between the CAD and non−CAD groups. Pooled odds ratios (ORs) with 95% confidence intervals (95%CIs) were estimated as pooled standard mean differences (SMD) with 95%CIs for consecutive variation. Heterogeneity was quantified by I^2^ statistic, where a value of 50% or greater indicated significant heterogeneity. When I^2^ > 50%, a random−effects model was chosen to pool the results, and when I^2^ < 50%, a fixed-effects model was used. A funnel plot test was used to detect publication bias. All statistical analyses were performed using Review Manager version 5.4.

## 3. Results

### 3.1. Study Selection and Quality Assessment

**Figure 1:**
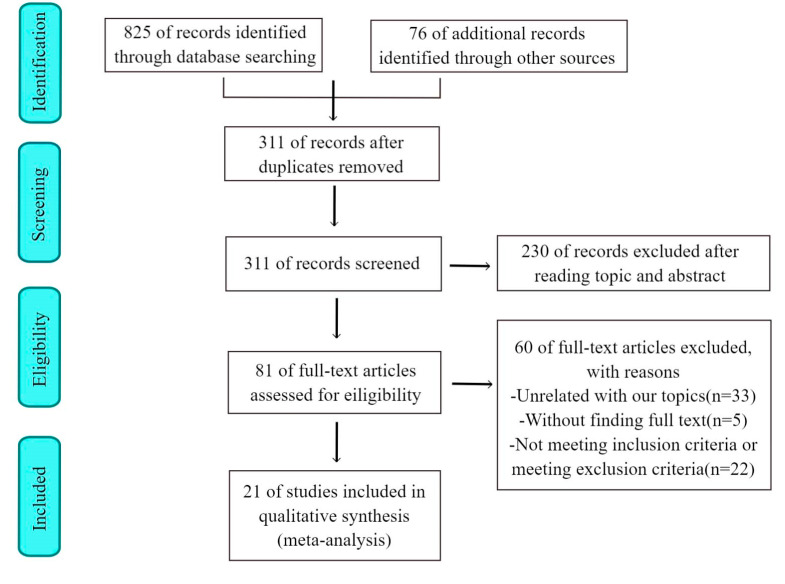
Flow chart of study selection in the meta-analysis.

According to the search strategy, 825 citations were obtained from the online database from 1 January 2000 to 1 May 2022. Seventy-six publications were included by manually searching the reference lists and reviewing the articles. After the removal of duplicates, 311 records remained in total. Then, 230 records were excluded by viewing titles and abstracts. Among the remaining 81 records, 60 citations were removed for various reasons. Finally, 21 full−text studies were suitable for this meta-analysis (Figure 1). The characteristics, quality evaluation, and demographics of the included studies are summarized in Table 1.

### 3.2. CAD Group versus Non-CAD Group

**Figure 2:**
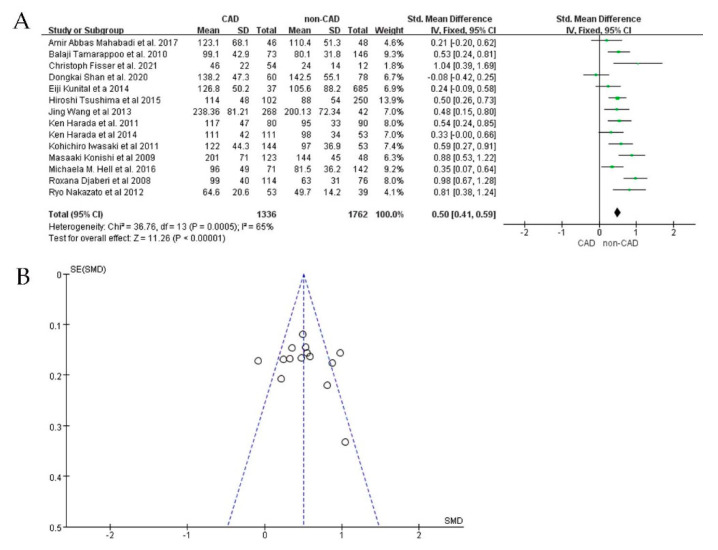
SMD by EAT volume between CAD patients and non−CAD patients. (**A**): Forest plot; (**B**): funnel plot.

**Figure 3:**
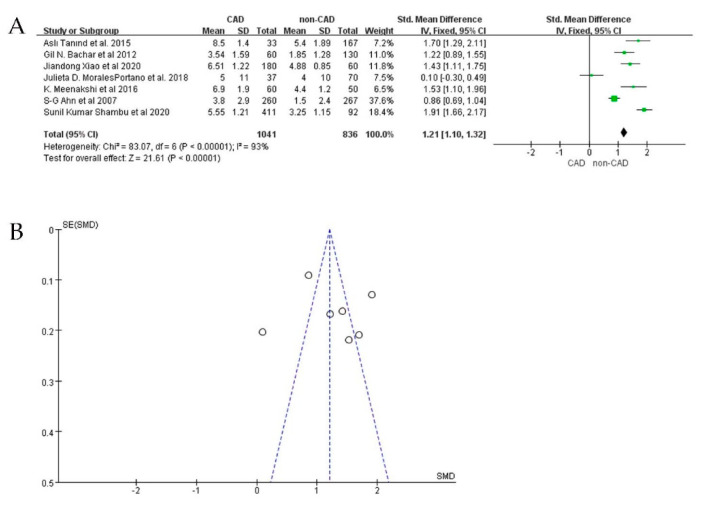
SMD by EAT thickness between CAD patients and non−CAD patients. (**A**) Forest plot; (**B**) funnel plot.

For all 21 studies reporting EAT in CAD, 14 of them detected the volume of EAT with CT. The volume of EAT in the CAD group (*n* = 1336) was significantly larger than that of the non-CAD group (*n* = 1762) (SMD: 0.50; 95%CI: 0.41, 0.59; I^2^ = 65%, *p* < 0.00001) (Figure 2A,B). In the other seven studies, the thickness of EAT detected by echocardiography was also significantly larger in the CAD group (*n* = 1041) than in the non−CAD group (*n* = 836) (SMD: 1.21; 95%CI: 1.10, 1.32; I^2^ = 93%; *p* < 0.00001) (Figure 3A,B).

**Figure 4:**
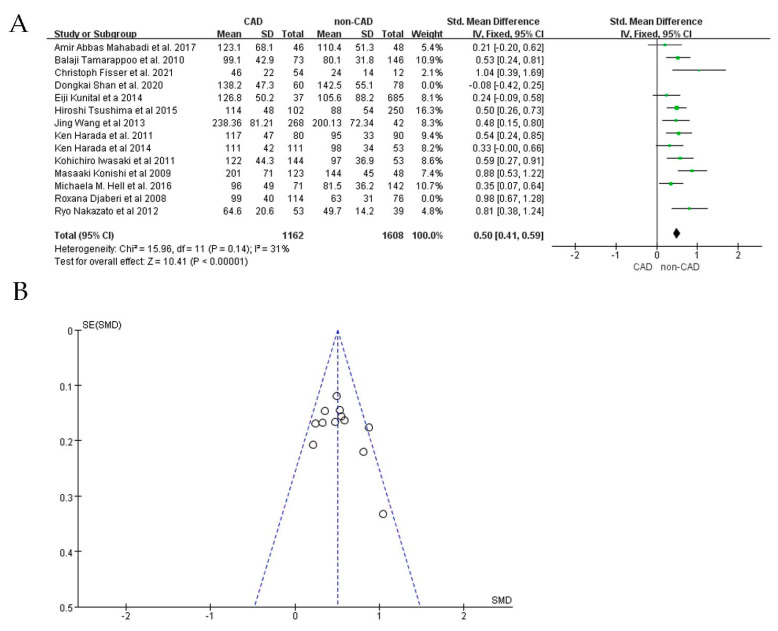
SMD of EAT volume between CAD patients and non−CAD patients after sensitivity analysis. (**A**): Forest plot; (**B**) funnel plot.

Analysis of the funnel plots of the two study groups (Figure 3A,B) revealed significant heterogeneity; the publication biases of the included studies may influence our meta-analysis results. We performed sensitivity analyses by removing individual studies one by one and performing additional meta−analyses with each study removed. We assessed the effect of each deletion on the pooled SMD. On the basis of the sensitivity analysis results, we observed that two studies interfered with the findings, making our meta−analysis statistically unstable [24,36]. When these two studies were excluded, the heterogeneity was significantly reduced (I^2^ = 31%; SMD: 0.50; 95%CI: 0.41, 0.59; *p* < 0.00001) (Figure 4A,B). Among studies measuring EAT thickness, a sensitivity analysis was performed. After removing each study, none of the studies were observed to affect the overall effect, suggesting that our meta-analysis was statistically stable.

### 3.3. Subgroup Analysis

**Figure 5:**
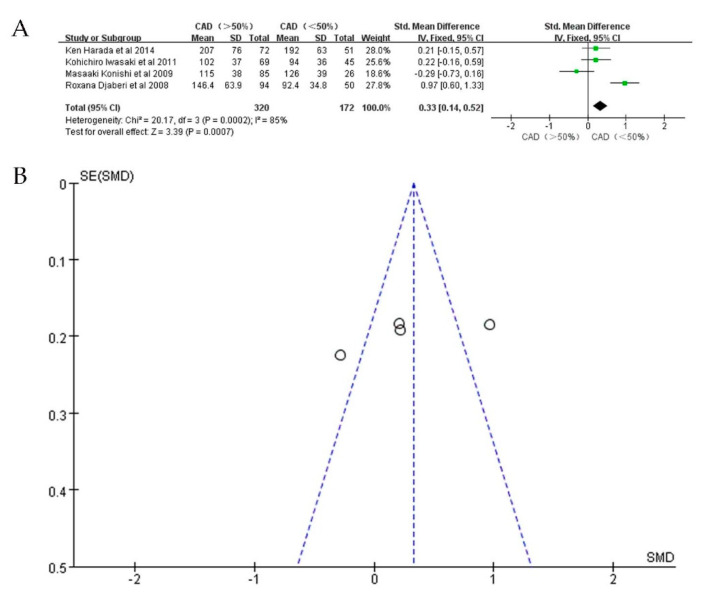
SMD of EAT volume between patients with severe CAD and mild / moderate CAD. (**A**): Forest plot; (**B**): funnel plot.

**Figure 6:**
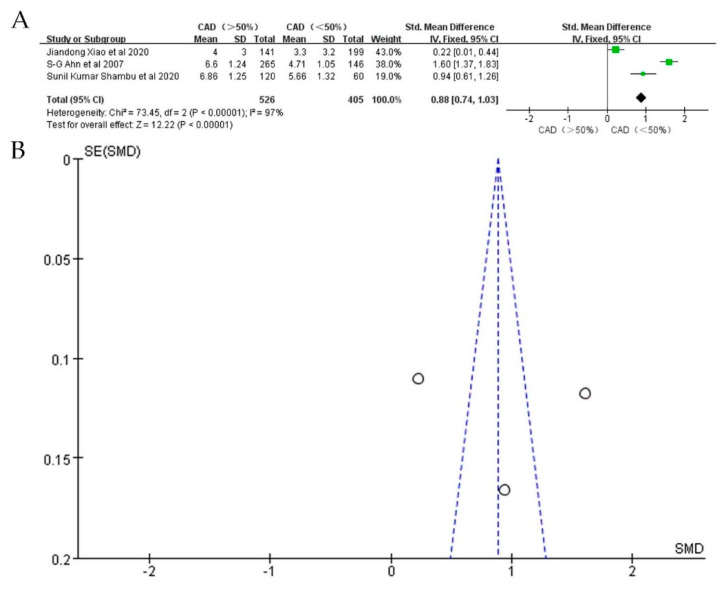
SMD of EAT thickness between patients with severe CAD and mild/moderate CAD. (**A**): Forest plot; (**B**): funnel plot.

To further investigate whether EAT is associated with CAD severity, we performed a subgroup analysis. Seven studies providing data on the relationship between EAT and CAD severity were included. In studies detecting EAT by CT, the volume of EAT in the severe CAD group (*n* = 320) was significantly larger than in the mild/moderate group (*n* = 172) (SMD: 0.33; 95%CI: 0.14, 0.52; I^2^ = 85%; *p* = 0.0007) (Figure 5A,B). Similarly, the thickness of EAT detected by echo was also significantly larger in the severe CAD group (*n* = 526) when compared with that of the mild/moderate group (*n* = 405) (SMD: 0.88; 95%CI: 0.74, 1.03; I^2^ = 97%; *p* < 0.00001) (Figure 6A,B).

**Figure 7:**
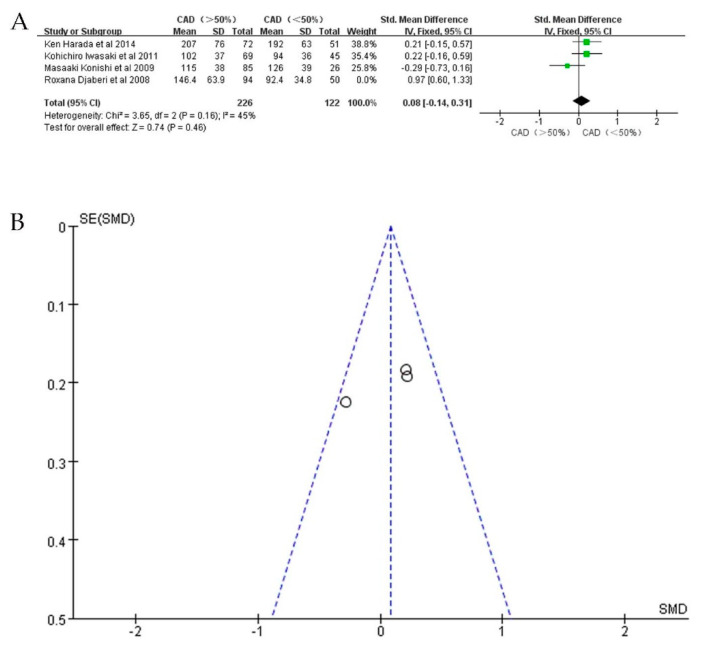
SMD of EAT volume between patients with severe CAD and mild/moderate CAD after sensitivity analysis. (**A**): Forest plot; (**B**): funnel plot.

In the subgroup analysis, we found high heterogeneity and performed a sensitivity analysis. In studies detecting EAT volume with CT, we observed one study (Mancio et al.) [24] that interfered with the findings (I^2^ = 85%). When this study was excluded, heterogeneity (I^2^ = 45%) was significantly reduced; however, the result was unable to achieve statistical significance (*p* = 0.46) (Figure 7A,B). This should mainly be attributed to the limited sample size. In studies detecting EAT thickness with echo, we were unable to perform a sensitivity analysis due to the limited sample size, and we could not determine whether EAT thickness was associated with CAD severity.

### 3.4. Publication Bias and Sensitivity Analysis 

Funnel plots indicated a symmetric distribution of the included studies. Sensitivity analyses confirmed the robustness of the results.

## 4. Discussion

This meta-analysis included 21 studies involving 1336 CAD patients and 1762 non-CAD patients. Our results showed that CAD patients tend to have larger EAT, supporting the association between enlarged EAT and the development of CAD. Moreover, we also concluded that patients with severe CAD have larger EAT than those with mild / moderate CAD when the high heterogeneity was not adjusted. After adjusting for study heterogeneity, several factors, such as different CAD types, EAT measure methods, and smaller sample sizes, may have led to insignificant differences. Thus, further studies with larger numbers, consistent measuring methods, and more specific CAD subtypes are required to confirm the association between EAT and CAD severity.

EAT is an extremely active adipose tissue with unique biological, molecular, and anatomical characteristics [63,64] that acts as an endocrine organ [7,19,65] to influence adjacent blood vessels through paracrine and endocrine signaling. EAT is capable of secreting inflammatory factors such as: TNFα [66], IL6 [67], adiponectin [68], leptin [69], etc. [11,70,71]. EAT is also a lipid storage unit and is actively involved in lipid metabolism and the energy homeostasis of the myocardium through the synthesis and release of FFAs [72]. Under physiological conditions, EAT exerts cardioprotective effects through its anti-atherosclerotic and anti−inflammatory properties, as well as high FFA release/uptake rates [73]. However, abnormally enlarged EAT will secrete a variety of bioactive substances as well as excess fatty acids, leading to systemic inflammation, insulin resistance, and dyslipidemia, which ultimately contribute to the development of atherosclerosis [74,75]. In recent years, research studying the relationship between EAT and CAD has gradually increased [12,76,77,78], but the reason for enlarged EAT in CAD patients remains unclear [30]. Therefore, we performed a meta-analysis to further explore the relationship between EAT and CAD.

According to our measurement methods, the properties of EAT were described as either CT−measured EAT volume or echo−measured EAT thickness. Regardless of measurement methods, our results showed that the volume and thickness of EAT in CAD patients are consistently larger than in non-CAD patients. To avoid heterogeneity between studies, we conducted a sensitivity analysis and excluded two studies [24,36]. Similar results were also achieved following adjustment. The main reasons for increased heterogeneity were as follow: (1) the methods of measuring and calculating EAT were not identical; (2) variation in the study populations and the classification of coronary obstruction severity; and (3) the comparison between ischemic and non-ischemic cardiomyopathy was unspecific with respect to the concept of CAD. In subgroup analyses, both the volume and the thickness of EAT were significantly larger in severe CAD patients (stenosis ≥ 50%) when compared with mild/moderate CAD patients. After adjusting heterogeneity, one study was excluded, but no significant difference was identified for EAT volume. However, due to limited samples, we could not conduct any further analyses of EAT thickness. Thus, our results suggested that enlarged EAT is associated with CAD severity, but this needs further verification in the future using a larger sample size.

The measurement methods of EAT include CT [16], echo [55], MRI [79], SPECT [80], and others, which can be used to measure the volume or thickness of EAT [8]. In normal hearts, EAT covers 80% of the surface [13,20]. The distribution of EAT is uneven, being more concentrated in the atrioventricular and interventricular grooves and around the epicardial coronary arteries. A smaller amount of EAT is found around the atria, over the free wall of the right ventricle, and on the apex of the left ventricle. Echo is currently the most convenient method of measuring EAT thickness, which is characterized by high repeatability and low cost [42,81,82,83]. However, echo cannot be used to perform the volumetric evaluation of EAT. The results of echo are highly dependent on the individual patient’s acoustic window, which may be suboptimal in obese patients due to fat impedance, leading to measurement errors [84]. CT is a simpler measurement method to measure the volume of EAT, with a higher spatial resolution providing a more accurate and reproducible quantification of EAT. It can also be used to quantify the volume of pericoronal adipose tissue (PCAT) and evaluate coronary arteries [85,86]. However, the result of CT is highly dependent on different standards in the attenuation range and errors caused by human factors between different observers and measurers. MRI and SPECT are used less frequently. In this study, a total of 14 studies measuring EAT volume by CT and 7 studies measuring EAT thickness by echo were included. Different measurement methods may bias the results of the analysis.

Consistent with previous studies, our study shows that, compared with non-CAD patients, the EAT of CAD patients is significantly enlarged, indicating that EAT may be involved in the occurrence of CAD, which may provide new ideas for further CAD research, diagnoses, and treatments and has certain clinical significance. However, the previous studies did not consider the influence of factors such as CAD classification and EAT measurement methods. Our findings suggest that EAT is consistently and abnormally elevated in CAD patients compared with non−CAD patients, even across different types of CAD or different EAT measurement methods, suggesting that abnormally enlarged EAT can serve as a predictor of CAD [17,78,87].

## 5. Limitations

Firstly, this is a retrospective study with a limited population, which limits our ability to compare the diagnostic accuracy of different subgroups. Secondly, the impact of potential factors such as blood lipids, BMI, and insulin resistance on the study should also be given consideration. In addition, this study focused on the quantitative comparison of EAT between CT and echo but did not consider the impact of the measurement location, method, or standard on EAT. Finally, the most important limitation is represented by the large heterogeneity among the studies. Although the heterogeneity was minimized with sensitivity analysis, it is still affected by the different standards of different experiments, so the results should be verified by prospective studies.

## 6. Conclusions

Our study shows that CAD patients have larger EAT compared to non−CAD patients, which provides a new approach for the diagnosis, treatment, and understanding of CAD. However, whether or not EAT can be used for the early assessment or diagnosis of CAD needs further confirmation. In general, our meta-analysis showed that patients with CAD have higher amounts of EAT than patients with non−CAD, regardless of CAD severity, multivessel stenotic CAD, or the EAT measurement methods used. This suggests that abnormally enlarged EAT may be involved in the development of CAD and is a useful predictor and potential therapeutic target for CAD.

## Figures and Tables

**Table 1:** Characteristics of included studies in the meta-analysis.

Author	Year	Country	Study Period	N	Male (%)	Age (yr)	Method	Expression	Study Type	Quality Score
Hiroshi Tsushima et al. [45]	2015	Japan	01/2008–04/2013	352	57	61 ± 11	CT	Volume	Retrospective	6
Jing Wang et al. [46]	2013	China	12/2006–01/2010	310	68.4	62.69 ± 10.78	CT	Volume	Retrospective	6
Masaaki Konishi et al. [47]	2009	Japan	04/2006–12/2008	171	59	60 ± 11	CT	Volume	Retrospective	7
Roxana Djaberi et al. [48]	2008	The Netherlands	NA	190	55	56 ± 12	CT	Volume	Retrospective	7
Ryo Nakazato et al. [49]	2012	USA	NA	92	68.5	71 ± 11	CT	Volume	Retrospective	8
Ken Harada et al. [50]	2014	Japan	01/2008–02/2009	164	70	65 ± 10	CT	Volume	Retrospective	6
Eiji Kunital et al. [51]	2014	Japan	11/2004–09/2009	722	61	65.0 ± 10.9	CT	Volume	Retrospective	6
Kohichiro Iwasaki et al. [52]	2011	Japan	06/2008–05/2009	197	62.4	65.1 ± 9.9	CT	Volume	Retrospective	6
Gil N. Bachar et al. [53]	2012	Israel	11/2007–01/2009	190	85.3	56.48 ± 9.2	CT	Thickness	Retrospective	6
K. Meenakshi et al. [54]	2016	India	NA	110	63.6	52.6 ± 0.6	Echo	Thickness	Retrospective	6
S-G Ahn et al. [55]	2007	Korea	NA	527	50.7	58 ± 11	Echo	Thickness	Prospective	8
Sunil Kumar Shambu et al. [34]	2020	India	02/2017–01/2019	503	36.6	59.8 ± 12.3	CT	Thickness	Retrospective	6
Jiandong Xiao et al. [28]	2020	China	05/2017–12/2018	240	95.4	56.66 ± 8.33	Echo	Thickness	Retrospective	6
Ken Harada et al. [56]	2011	Japan	2009–2010	170	77	65 ± 12	CT	Volume	Prospective	6
Amir Abbas Mahabadi et al. [57]	2017	Germany	2010–2015	94	60.6	66.9 ± 14.7	CT	Volume	Retrospective	6
Michaela M. Hell et al. [58]	2016	Germany	2002–2008	213	90	60 ± 10	CT	Volume	Prospective	7
Balaji Tamarappoo et al. [59]	2010	American	NA	219	90.4	60.3 ± 10.4	SPECT	Volume	Prospective	7
Christoph Fisser et al. [60]	2021	Germany	NA	66	83.3	55 ± 10	CT	Volume	Prospective	6
Dongkai Shan et al. [61]	2020	China	2012–2016	138	66.7	61.7 ± 8.9	CT	Volume	Retrospective	8
Julieta D. MoralesPorta et al. [31]	2018	Korea	2013–2016	107	80.4	63.6 ± 9.67	Echo	Thickness	Prospective	8
Aslı Tanınd et al. [62]	2015	Turkey	2012–2012	200	80	63 ± 13	Echo	Thickness	Prospective	8

## Data Availability

Data can be obtained by contacting the corresponding author.
